# Review of Research Into Employer Approaches to Younger Onset Dementia and Mild Cognitive Impairment in the Workplace

**DOI:** 10.1093/geroni/igab046.1879

**Published:** 2021-12-17

**Authors:** James Carino, Philip Taylor, Damian Morgan

**Affiliations:** 1 Federation University Australia, Berwick, Victoria, Australia; 2 Federation University Australia, Churchill, Victoria, Australia

## Abstract

Younger Onset Dementia (YOD) and Mild Cognitive Impairment (MCI) are relatively prevalent conditions globally which can affect the job performance of individuals in their working lives. This presentation considers the existing research documenting actual, stated or intended approaches taken by employers to managing and supporting employees with these conditions. Nine relevant research projects were identified based on an extensive exploration of the peer reviewed literature. These show that employers have some knowledge of dementia, but do not recognise this as a possible explanation when performance changes occur in the workplace. Employees typically leave or are removed from the workplace before a formal diagnosis or soon after. The literature shows both supportive and unsupportive behaviours toward employees. Drawing from this literature recommendations for increasing the quality of support provided to employees are offered: awareness training to foster earlier detection, clarification of these conditions as a disability, and application of methods to support employees to continue to contribute to the workplace while this is feasible and desirable from both the employer and the employee’s perspective.

